# Diagnostic Yield and Utility of Radiographic Imaging in the Evaluation of Pulsatile Tinnitus: A Systematic Review

**DOI:** 10.1097/ONO.0000000000000030

**Published:** 2023-05-12

**Authors:** Austin C. Cao, Tiffany P. Hwa, Caitlin Cavarocchi, Alexandra Quimby, Steven J. Eliades, Michael J. Ruckenstein, Douglas C. Bigelow, Omar A. Choudhri, Jason A. Brant

**Affiliations:** 1 Department of Otolaryngology-Head and Neck Surgery, Hospital of the University of Pennsylvania, Philadelphia, PA; 2 Department of Radiology, Hospital of the University of Pennsylvania, Philadelphia, PA.

**Keywords:** Diagnosis, Imaging, Meta-analysis, Pulsatile tinnitus, Systematic review

## Abstract

**Objective:**

The objective of this study is to assess diagnostic yield of imaging modalities used to evaluate patients presenting with pulsatile tinnitus (PT).

**Databases Reviewed:**

PubMed, Embase, and Scopus were queried using the search terms “pulsatile tinnitus,” “pulse-synchronous tinnitus,” and “pulse synchronous tinnitus” with no date limitations.

**Methods:**

Studies that reported diagnostic imaging for patients presenting with PT were included. Data were reviewed for sample size, gender, age, imaging study, indications, and diagnoses. The primary outcome measure from aggregated data was the yield of positive diagnoses made with each imaging modality. The quality of evidence was assessed for risk of bias.

**Results:**

From an initial search of 1145 articles, 17 manuscripts met inclusion criteria, of which 12 studies evaluated individual imaging modalities. The number of unique patients included was 1232. The diagnostic yield varied between modalities: carotid ultrasound (21%, 95% confidence interval [CI]: 12%–35%), CT temporal bone (65%, CI: 20%–93%), computed tomographic angiography (86%, CI: 80%–90%), and MRI/magnetic resonance angiography (58%, CI: 43%–72%).

**Conclusion:**

Studies on the diagnostic approach to PT are limited by heterogeneity in both inclusion criteria and reporting standards. A wide range of imaging modalities are used in practice during the initial evaluation of PT, and the diagnostic yield for imaging can be improved by utilizing more specific clinical indications.

Pulsatile tinnitus (PT), or the perception of a rhythmic or pulsing sound synchronized with the heart beat, is an uncommon, but often bothersome symptom that can arise from a wide range of underlying pathologies. The differential diagnosis for PT includes both vascular and nonvascular entities, as well as benign and potentially life-threatening diagnoses (1). Although clinical examination can occasionally yield informative findings such as an audible bruit or the visualization of a vascular middle ear mass, patients will often present with a normal clinical examination and audiometric testing despite persistent symptoms.

Prior literature has demonstrated that with an appropriate diagnostic evaluation, a causative clinical entity for PT may be identified in approximately 70% of patients (2). In light of this information, and the onus on providers to identify potential life-threatening conditions, clinicians would benefit from a systematic diagnostic approach to the assessment of PT (1–4). This diagnostic approach must be broad enough to encompass diagnoses as disparate as superior semicircular canal dehiscence, carotid artery stenosis, and intracranial arteriovenous malformations, among others.

To that end, numerous investigative efforts have sought to evaluate the diagnostic yield of specific imaging modalities or to propose a multimodal diagnostic algorithm. In many of these cases, the yield of a particular diagnostic imaging test has only been evaluated for specific disease entities. However, for a given patient presenting with undiagnosed PT, there remains no evidence-based consensus on the optimal approach to diagnostic work-up. In this report, we present our results from a systematic review of the literature and resultant analysis evaluating the diagnostic yield of various imaging studies for patients presenting with PT.

## METHODS

This study was exempt from review in keeping with policies of the Hospital of the University of Pennsylvania Institutional Review Board.

### Search Methodology

A systematic review was performed. A literature query was undertaken on December 1, 2020, using three databases (PubMed, Embase, and Scopus), employing a Boolean phrase search strategy. The search did not impose a publication date limit. All English publications in the databases were queried using the following search terms: “pulsatile tinnitus” OR “pulse-synchronous tinnitus” OR “pulse synchronous tinnitus.” The inclusion of studies along these terms was evaluated using the PubMed Database’s MeSH term definitions.

Following removal of duplicates, 2 reviewers from our group (A.C.C.) and (J.A.B.) screened the titles and abstracts from the search results using the following criteria: 1) papers had to include patients under evaluation for a presenting symptom of PT; and 2) papers had to reference diagnostic evaluation of PT.

**FIG. 1.**
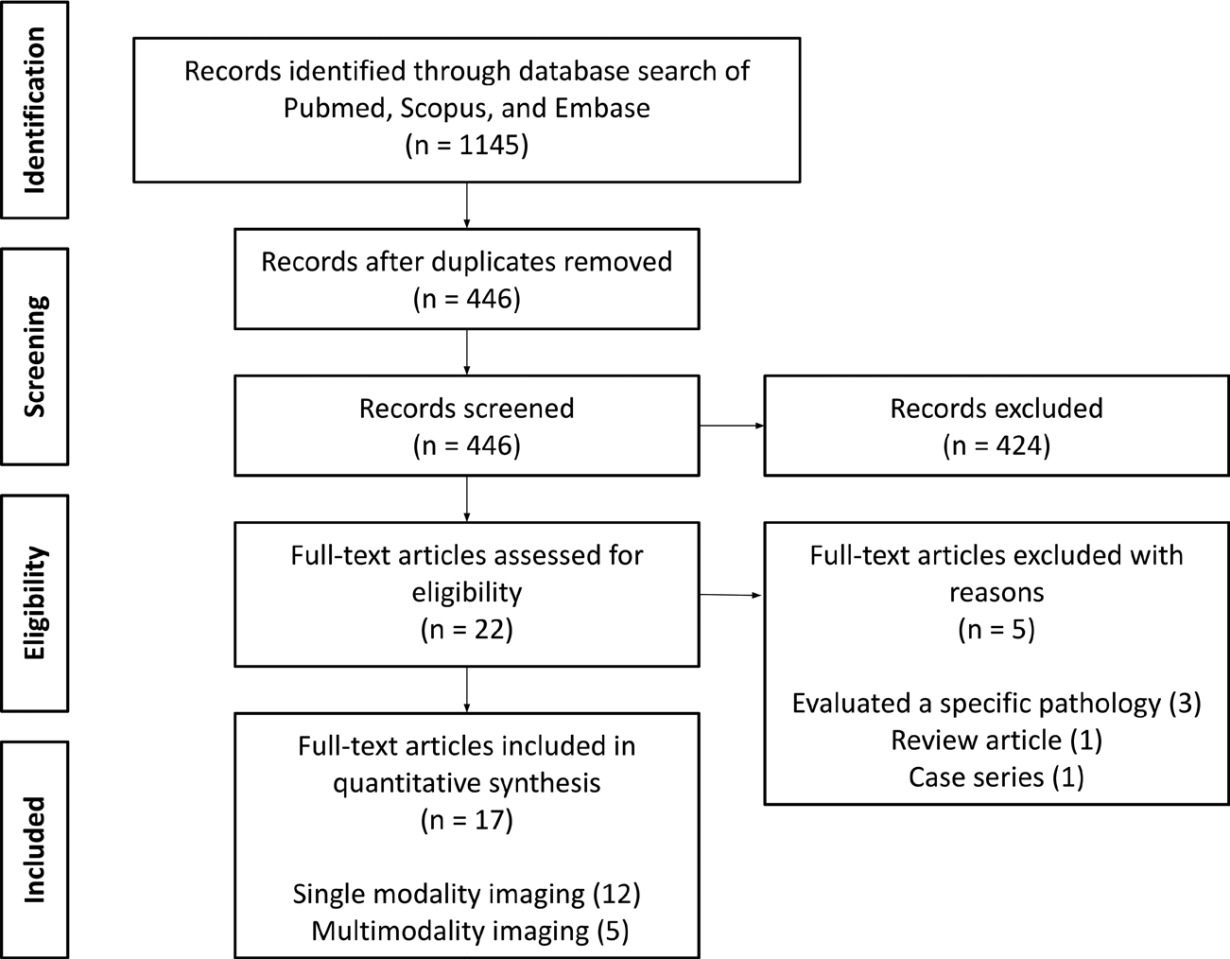
Preferred Reporting Items for Systematic Reviews and Meta-Analyses diagram describing study selection.

In the case of any discrepancies between the initial 2 reviewers, a third reviewer (T.P.H.) reviewed the discrepancies in a blinded fashion and acted as the deciding opinion. Before reviewing full article texts, the references of included articles were screened for additional articles appropriate for review. Data extracted from full-text review of studies included the diagnostic imaging or studies, clinical consultations for diagnostic work-up, results of diagnostic work-up, and diagnostic yield. The literature review, search methodology, and findings are presented using Preferred Reporting Items for Systematic Reviews and Meta-Analyses guidelines and outlined in Figure 1.

### Statistical Analysis

Statistical analyses were performed with RStudio (Boston, MA). Results were notable for wide variability in reporting and differences in inclusion criteria and imaging criteria for a given modality across studies, as well as a lack of reporting on multimodality results at the level of the individual patient. Furthermore, due to duplication of individual patients across results in multimodal studies, further patient-level or study-wide modality-specific statistical analyses could not be performed.

Consequently, our analysis was limited to the evaluation of pooled diagnostic yields and assessment of each imaging modality with a random effects model where possible. These results are further represented as forest plots. For studies that evaluated multiple modalities within a single cohort of patients, subjects were duplicated to calculate the pooled diagnostic yield of each individual diagnostic modality. A risk-of-bias assessment was performed by a single reviewer (Supplemental Table 1, http://links.lww.com/ONO/A9).

## RESULTS

### Results of Systematic Review

Initial Boolean search using the previously detailed commands of three database (PubMed, Embase, Scopus) yielded 1143 results. After removal of 699 duplicates, initial review of title and abstract by 2 independent authors included 19 studies. Five discrepancies were then impartially reviewed by a third reviewer, 3 of which were included, for a final yield of 22 studies included from the initial review.

The full text of these 22 studies were reviewed, of which 5 were not included in the final analysis due to the following reasons: evaluation of a specific pathology rather than chief complaint of PT (n = 3), review article (n = 1), or case series (n = 1) (Fig. 1). All full texts were in English. Publication dates of the 17 included studies ranged from 1990 to 2018, and are presented in summative fashion, categorized by modality of imaging evaluated, alongside sample size, subject cohort details, relevant inclusion criteria, and overall diagnostic yield of the modalities under investigation (Table 1) (2–18).

**TABLE 1.** Evidence table showing clinical and design characteristics of individual studies

Author, year	Design	Data collection	Modality	Patients	Age	Sex (female)	Diagnostic yield	Additional details
Studies evaluating multiple imaging modalities								
Remley e al (3), 1990	Cohort	Retrospective	Multimodal	107	7–88	78	80.4%	Test battery at discretion of provider
			CT temporal bone	69	-	-	81.2%	
			CTA	68	-	-	83.8%	
			MRI	24	-	-	79.2%	
Mattox and Hudgins (2), 2008	Cohort	Retrospective	Multimodal	54	-	38	72.2%	Test battery at discretion of provider. Excluded chemodectomas
Sismanis (4), 1998	Cohort	Retrospective	Multimodal	145	-	-	91.0%	Test battery at discretion of provider
Sonmez et al (5), 2007	Cohort	Retrospective	Multimodal	74	42.8, 5–97	30	67.6%	Test battery at discretion of provider
Waldvogel et al (6), 1998	Cohort	Retrospective	Multimodal	84	51, 3–82	58	67.9%	Test battery at discretion of provider
			CDS	40	-	-	30.0%	
			CTA	36	-	-	97.2%	
			Cranial CT	14	-	-	21.4%	
			MRI/MRA	12	-	-	41.7%	
Studies comparing imaging modalities								
Deuschl et al (7), 2015	Cohort	Retrospective	DSA	54	51.50	37	68.5%	Patient included if MRI and DSA were both performed at discretion of provider
			MRI/MRA	54	51.50	37	68.5%	
Mohseni et al (8), 2018	Case–control	Prospective	CTA	30	50.83, SD 11.26	20	73.3%	Patients included if subjective PT only with normal examination
			CT temporal bone	30	50.83, SD 11.26	20	36.7%	
Shweel and Hamdy (9), 2013	Cohort	Retrospective	MRI/MRA	27	23–85	16	40.7%	Patients included if normal examination
Tsai et al (10), 2016	Cohort	Prospective	CDS	155	53.35	113	18.1%	N/A
Studies evaluating single imaging modalities								
Dietz et al (11), 1994	Cohort	Retrospective	MRI/MRA	49	48, 12–80	31	57.1%	N/A
Dong et al (12), 2015	Cohort	Retrospective	CT temporal bone	242	40.9, 11–69	215	70.2%	Patients included if venous origin on examination
Krishnan et al (13), 2006	Cohort	Prospective	CTA	16	49, 27–73	9	43.8%	Patients included if CTA/V was performed at discretion of provider, with normal examination
In ‘t veld et al (14), 2018	Cohort	Prospective	DSA	50	53, SD 13	27	30.0%	Patients included if DSA performed to exclude AV shunt
Mundada et al (15), 2015	Cohort	Retrospective	CTA	32	15–74	20	96.9%	Patients included if CT A/V performed at discretion of provider, with normal examination
Sanchez et al (16), 1998	Cohort	Prospective	MRA	16	42.5, 25-71	15	81.3%	Patients included if normal exam.
Shin et al (17), 2000	Cohort	Retrospective	MRI/MRA	33	51, 22–87	20	69.7%	Patients included if MRI/MRA was performed at discretion of provider
Terzi et al (18), 2015	Case–control	Prospective	CDS	34	53.00	22	23.5%	N/A

CDS indicates carotid Doppler study; CTA, computed tomographic angiography; DP-CECT, dual-phase contrast-enhanced CT; DSA, digital subtraction angiography; MRA, magnetic resonance angiography; PT, pulsatile tinnitus.

Studies varied widely in both the imaging modality or modalities of investigative interest and in the approach for diagnostic imaging. Eight studies reviewed the diagnostic yield of a single imaging modality, and 4 studies compared the diagnostic yield between imaging modalities. The remaining 5 studies evaluated a multimodal test battery. Seven studies included imaging as ordered at the discretion of the provider without specified criteria, and 7 studies had specific clinical requirements for inclusion, such as a normal clinical examination or clinical findings suggestive of venous origin. The majority of included studies were retrospective in design, with 6 prospective studies reviewed. Included studies met a median of 5/6 criteria on the risk-of-bias assessment, and three studies only met 3/6 criteria. All 3 of these studies evaluated multimodal test batteries and did not report diagnostic yields for individual modalities.

### Analysis of Diagnostic Yield

**FIG. 2.**
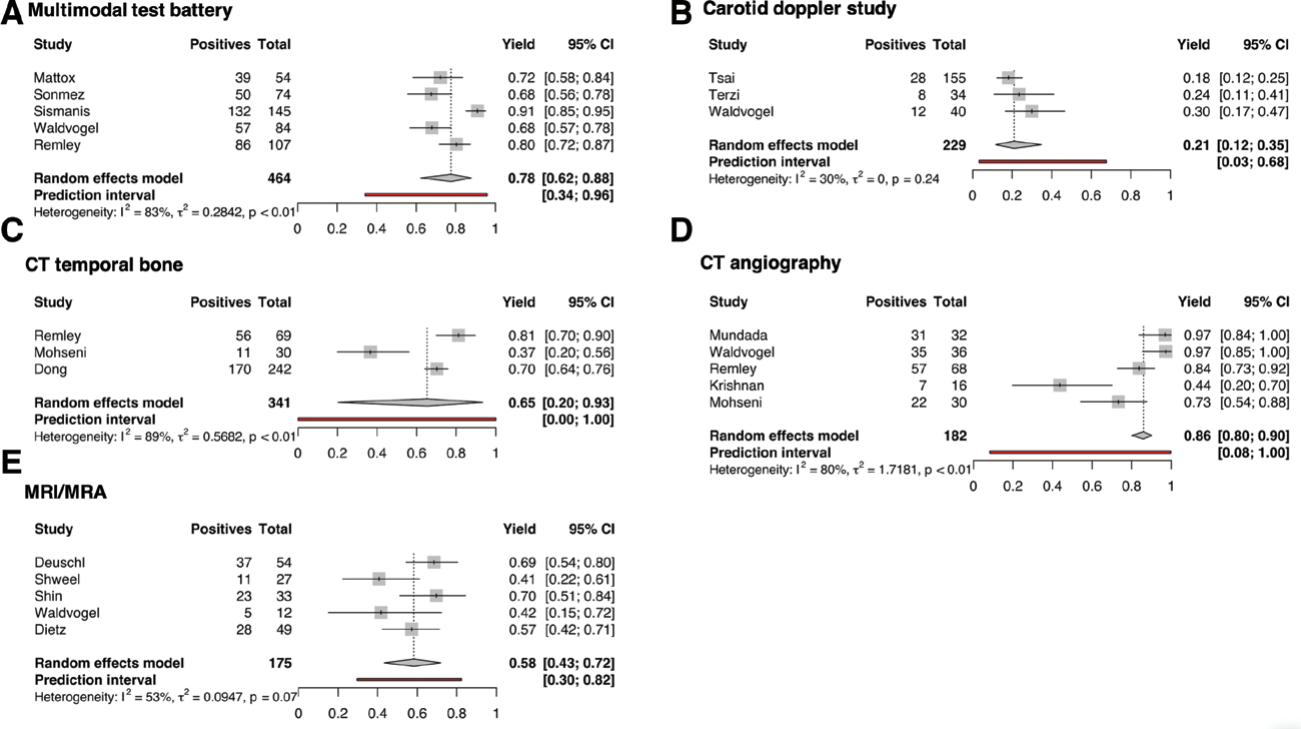
Forest plots of diagnostic yield by imaging technique. Each subfigure includes all studies that report evaluating (*A*) multimodal test battery, (*B*) carotid Doppler study, (*C*) CT temporal bone, (*D*) CT angiography, and (*E*) MRI/MR angiography. CTA indicates computed tomography.

In light of differences in modality of interest, inclusion criteria, and study design, the overall diagnostic yield was highly variable and ranged from 24% to 97% across all reported modalities (2–18). After pooling of results across studies, diagnostic yield varied from a low of 21% (95% confidence interval [CI]: 12%–35%) in carotid duplex sonography (CDS) to a high of 86% (CI: 80%–90%) for computed tomographic angiography (CTA). Diagnostic yield for MRI and magnetic resonance angiography (MRA) was 58% (CI: 43%–72%). Studies evaluating a multimodal test battery resulted in a yield of 78% (CI: 62%–88%). The complete results of pooled diagnostic yield are summarized in Table 2. We additionally performed a random effects model on each of the imaging modalities (Fig. 2). Moderate to high heterogeneity was found in subgroups, ranging from 30% in carotid ultrasound to 89% in CT temporal bone. Digital subtraction angiography (DSA) could not be assessed with random effects modeling due to insufficient studies evaluating this modality in isolation (7,9). Additionally, the individual contributions of MRI and MRA could not be assessed, since studies evaluated the diagnostic yield of MRI/MRA in conjunction rather than as independent modalities (6,7,9,11,17).

**TABLE 2.** Pooled diagnostic yield by imaging technique

Imaging	Studies	Patients	Diagnostic positives	Diagnostic yield (random effects model)	Lower 95% CI	Upper 95% CI
CDS	3	229	48	0.21	0.12	0.35
CT temporal bone	3	341	237	0.65	0.2	0.93
CTA	5	182	152	0.86	0.8	0.9
MRI/MRA	5	175	104	0.58	0.43	0.72
DSA	2	104	52	0.49	0	1
Multimodal	5	464	364	0.78	0.62	0.88

CDS indicates carotid Doppler study; CTA, computed tomographic angiography; DSA, digital subtraction angiography; MRA, magnetic resonance angiography.

## DISCUSSION

### Literature Review for the Diagnostic Evaluation of PT

We report the first systematic review of the literature evaluating the diagnostic yield of imaging modalities for the evaluation of PT. The potential etiologies of PT span a wide breadth of anatomical region and pathophysiology. This oft-bothersome symptom may be caused by atherosclerotic carotid disease, fibromuscular dysplasia, intracranial vascular anomalies, glomus tumors of the jugular bulb and middle ear, idiopathic or tumor-related causes of elevated intracranial pressure, or defects of the skull base including dehiscence of the superior semicircular canal or sigmoid sinus (1,2). Because the work-up of PT engages a multidisciplinary cohort of clinicians, our approach of using a systematic review is critical to achieve an adequately thorough survey of the existing literature across medical subspecialties for the assessment of overall yield.

In our analysis, CTA demonstrated the highest overall diagnostic yield on pooled analysis and random effects modeling. Importantly, the pooled yields were equivalent for CTA versus multimodal test battery, although it should be noted that multimodal studies were generally older and did not always include specific modalities like CT angiography. Of the 3 studies evaluating multimodal test battery that did not report diagnostic yield for individual modalities, the modalities used were as follows: Mattox and Hudgins (2) (carotid ultrasound, CTA, MRI/MRA, cerebral arteriography), Sismanis (4) (CT temporal bone, carotid angiography, carotid ultrasound, and MRI/MRA), and Sonmez et al (5) (CT temporal bone, carotid ultrasound, CT brain, MRI/MRA, DSA). Two out of 5 multimodal studies did not utilize CTA imaging during diagnostic workup, which may partially account for the lower overall diagnostic yield in these studies. In a retrospective cohort of 255 patients at our institution, an overall diagnostic yield of 85% was found, higher than the pooled yield in this meta-analysis (unpublished). Most notably, the heterogeneity of existing literature precluded formal cross-modality analysis due to significant differences in inclusion criteria, indications for imaging, and outcomes reporting. Due to these differences, there is also a paucity of robust evidence to generate an evidence-based approach to imaging work-up based on clinical findings. For example, only 1 study utilized inclusion criteria based on specific clinical exam findings—Dong et al (12) studied patients specifically presenting with suggested venous origin on examination. Consequently, we seek to summarize the different diagnostic approaches for PT presented in the literature and discuss notable divergence points for further investigation.

On initial assessment by an otolaryngologist, patients undergo a thorough history, physical examination, and audiometric evaluation. Focused clinical assessment is performed with the intent to determine whether the underlying pathology may be arterial or venous in origin. Physical examination maneuvers to assist in this include the impact of ipsilateral versus contralateral compression of the internal jugular vein and head rotation on symptomatology (suggestive of venous pathology) and assessing for bruits (suggestive of arterial pathology).

In patients presenting with a carotid bruit, carotid Doppler sonography is a low-cost screening tool often recommended by clinicians, despite having a pooled diagnostic yield of 21% across the literature (6,10,18). Based on data reported by Waldvogel and subsequently confirmed by Tsai et al, CDS has high specificity for cervical carotid pathology compared to MRI, MRA, CT, and CTA (6,10). In their study, 25 of 25 diagnoses identified on CDS were subsequently confirmed. However, among 52 subjects with normal CDS, 13 diagnoses were subsequently found on further work-up. Finally, an audible bruit over the orbit or periauricular area always prompts further evaluation with noninvasive angiography (1,2,19).

If a middle ear mass or bony dehiscence of vascular canals is suspected on clinical examination, institutional studies suggest that initial evaluation with CT temporal bone is sufficient (1,2,19,20). Some authors argue for the broader use of CT angiography in place of a limited scan, as the examination findings may be nonspecific and CTA may yield less common venous pathologies (8,15,19). In particular, Mohseni et al (8) reports a prospective case–control study in which CTA captured 22 of 30 diagnoses compared with 11 of 30 diagnoses captured by CT temporal bone alone. Bone window CT reconstructions can be obtained from CTA/V to diagnose middle ear or temporal bone pathologies. Mattox and Hudgins (2) favors use of CTA/V for initial imaging of PT of venous origin due to superior visualization of vasculature and the ability to evaluate bony anatomy.

More recent studies also support the use of MRI/MRA as an equivalent imaging tool when noninvasive angiography is indicated (9,21). In a cohort of 27 patients, Shweel and Hamdy (9) reported 80% sensitivity and 88% specificity for combined MRI/MRA when compared with the test battery. Furthermore, Deuschl et al (7) reported identical findings between MRI/MRA and DSA in 54 patients. As its availability and accessibility has grown in the United States, MRI/MRA has distinct advantages of decreasing radiation exposure and providing higher contrast resolution. Although the pooled diagnostic yield for MRI/MRA was lower than CTA in this study, it should be noted that 3 of the 5 studies included in the MRI/MRA group were older than 20 years and may not reflect recent advances in MRI technology.

### Limitations and Future Directions

There are several noteworthy limitations to this study. First, cross-modality statistical comparisons in this investigation were limited by heterogeneity in the literature. This heterogeneity was attributable to differences in study design, inclusion criteria, and the diagnostic studies of interest. Importantly, the use of specific imaging modalities over time and recent improvements in imaging technology increased the heterogeneity within pooled datasets. There were also notable differences in reporting and analysis, with few studies providing the patient-level data that would allow for comparisons with meta-analysis. For multimodal imaging studies, 3 of the 5 studies reported the overall diagnostic yield for their proposed test battery, but they did not include the breakdown for individual imaging tests. Furthermore, multimodal diagnostic estimates are limited to these studies, and more consistent testing of a variety of testing algorithms will be necessary to evaluate yield and accuracy in practice. The literature reviewed in this report spans multiple medical disciplines, which may have further contributed to differences in reporting.

To design a diagnostic algorithm, future study should be directed at formal evaluation of imaging modalities in a prospective fashion. Few studies compared the findings from each imaging modality with the final diagnosis, contributing to error when the underlying etiology differs from imaging findings. To improve the data set in the literature on diagnostic yield in the assessment of PT, we recommend a minimum reporting standard for imaging in PT that includes both modality-specific diagnostic yields and outcomes.

## CONCLUSIONS

We present a systematic review of imaging modalities used in the workup of undifferentiated PT. Initial assessment decisions should be tailored for suspected pathology based on clinical assessment. Current literature evaluating diagnostic yield in the imaging work-up of PT is limited by variability in inclusion criteria and limited head-to-head comparisons of different imaging modalities. Nonetheless, the positive yield for imaging can be improved by utilizing specific indications during the diagnostic workup of PT.

## FUNDING SOURCES

None declared.

## CONFLICT OF INTEREST STATEMENT

None declared.

## DATA AVAILABILITY STATEMENT

This study was exempt from review in keeping with policies of the Hospital of the University of Pennsylvania Institutional Review Board. The review was not registered. All study data and analytic code can be made available by contacting the corresponding author.

## Supplementary Material



## References

[1] Sismanis A. Pulsatile tinnitus: contemporary assessment and management. Curr Opin Otolaryngol Head Neck Surg. 2011;19:348–357. 22552697 10.1097/MOO.0b013e3283493fd8

[2] Mattox DE Hudgins P. Algorithm for evaluation of pulsatile tinnitus. Acta Otolaryngol. 2008;128:427–431. 18368578 10.1080/00016480701840106

[3] Remley KB Coit WE Harnsberger HR Smoker WR Jacobs JM McIff EB. Pulsatile tinnitus and the vascular tympanic membrane: CT, MR, and angiographic findings. Radiology. 1990;174:383–389. 2296650 10.1148/radiology.174.2.2296650

[4] Sismanis A. Pulsatile tinnitus a 15-year experience. Am J Otol. 1998;19:l998. 9661757

[5] Sonmez G Basekim CC Ozturk E Gungor A Kizilkaya E. Imaging of pulsatile tinnitus: a review of 74 patients. Clin Imaging. 2007;31:102–108. 17320776 10.1016/j.clinimag.2006.12.024

[6] Waldvogel D Mattle HP Sturzenegger M Schroth G. Pulsatile tinnitus—a review of 84 patients. J Neurol. 1998;245:137–142. 9553842 10.1007/s004150050193

[7] Deuschl C Göricke S Gramsch C. Value of DSA in the diagnostic workup of pulsatile tinnitus. PLoS One. 2015;10:e0117814. 25689158 10.1371/journal.pone.0117814 PMC4331557

[8] Mohseni M Asghari A Daneshi A Jalessi M Rostami S Nasoori Y. Comparison of brain CT angiography/venography and temporal bone HRCT scan findings in patients with subjective pulsatile tinnitus in affected side and unaffected side. Biomed Res Ther. 2018;5:2455–2465.

[9] Shweel M Hamdy B. Diagnostic utility of magnetic resonance imaging and magnetic resonance angiography in the radiological evaluation of pulsatile tinnitus. Am J Otolaryngol. 2013;34:710–717. 24041839 10.1016/j.amjoto.2013.08.001

[10] Tsai LK Yeh SJ Tang SC. Validity of carotid duplex sonography in screening for intracranial dural arteriovenous fistula among patients with pulsatile tinnitus. Ultrasound Med Biol. 2016;42:407–412. 26614386 10.1016/j.ultrasmedbio.2015.10.013

[11] Dietz RR Davis WL Harnsberger HR Jacobs JM Blatter DD. MR imaging and MR angiography in the evaluation of pulsatile tinnitus. Am J Neuroradiol. 1994;15:879–889. 8059655 PMC8332180

[12] Dong C Zhao PF Yang JG Liu ZH Wang ZC. Incidence of vascular anomalies and variants associated with unilateral venous pulsatile tinnitus in 242 patients based on dual-phase contrast-enhanced computed tomography. Chin Med J (Engl). 2015;128:581–585. 25698187 10.4103/0366-6999.151648 PMC4834766

[13] Krishnan A Mattox DE Fountain AJ Hudgins PA. CT arteriography and venography in pulsatile tinnitus: preliminary results. Am J Neuroradiol. 2006;27:1635–1638. 16971601 PMC8139805

[14] In ‘t veld M Fronczek R de Laat JA Kunst HP Meijer FJ Willems PW. The incidence of cranial arteriovenous shunts in patients with pulsatile tinnitus: a prospective observational study. Otol Neurotol. 2018;39:648–653. 29561378 10.1097/MAO.0000000000001767

[15] Mundada P Singh A Lingam RK. CT arteriography and venography in the evaluation of pulsatile tinnitus with normal otoscopic examination. Laryngoscope. 2015;125:979–984. 25379666 10.1002/lary.25010

[16] Sanchez TG Santoro PP Torres de Medeiros IR Bittar RS Bento RF. Magnetic resonance angiography in pulsatile tinnitus: the role of anatomical variations. Int Tinnitus J. 1998;4:122–126. 10753399

[17] Shin EJ Lalwani AK Dowd CF. Role of angiography in the evaluation of patients with pulsatile tinnitus. Laryngoscope. 2000;110:1916–1920. 11081610 10.1097/00005537-200011000-00028

[18] Terzi S Arslanoğlu S Demiray U Eren E Cancuri O. Carotid Doppler ultrasound evaluation in patients with pulsatile tinnitus. Indian J Otolaryngol Head Neck Surg. 2015;67:43–47. 25621231 10.1007/s12070-014-0756-9 PMC4298576

[19] Pegge S Steens S Kunst H Meijer F. Pulsatile tinnitus: differential diagnosis and radiological work-up. Curr Radiol Rep. 2017;5:5. 28203490 10.1007/s40134-017-0199-7 PMC5263210

[20] Wang A Nelson A Pino C Svider P Hong R Chan E. Management of sigmoid sinus associated pulsatile tinnitus: a systematic review of the literature. Otol Neurotol. 2017;38:1390–1396. 29135862 10.1097/MAO.0000000000001612

[21] Grierson K Bou-Haidar P Dumper J Fagan P. The assessment of pulsatile tinnitus—a systematic review of underlying pathologies and modern diagnostic approaches. Aust J Otolaryngol. 2018;1:27.

