# Supplementary material for: Diagnostic Yield and Utility of Radiographic Imaging in the Evaluation of Pulsatile Tinnitus: A Systematic Review
Source: Otol Neurotol Open. 2023 May 12;3(2):e030. doi: 10.1097/ONO.0000000000000030 (PMC10950154; doi:10.1097/ONO.0000000000000030)
Supplement: Supplementary file 1 [file ono-3-e030-s001.pdf]

Author of Study	Is the Aim/Objective of the Study Clearly Described?	Are the Main Outcomes To Be Measured Clearly Described in the Introduction or Methods?	Are the Characteristics of the Patients Included in the Study Clearly Described?	Is the Procedure of Interest Clearly Described?	Are the Main Findings of the Study Clearly Described?	Were the Subjects Asked To Participate in the Study Representative of the Entire Population from which They Were Recruited?
**Studies evaluating multiple imaging modalities**						
Remley	Yes	Yes	Yes	Yes	Yes	Yes
Mattox	Yes	Yes	No	No	No	Yes
Sismanis	Yes	Yes	No	No	No	Yes
Sonmez	Yes	No	Yes	No	No	Yes
Waldvogel	Yes	Yes	Yes	Yes	Yes	Yes
**Studies comparing imaging modalities**						
Deuschl	Yes	Yes	No	Yes	Yes	Unclear
Mohseni	Yes	Yes	Yes	Yes	Yes	Yes
Shweel	Yes	Yes	Yes	No	Yes	Yes
Tsai	Yes	Yes	No	Yes	Yes	Yes
**Studies evaluating single imaging modalities**						
Dietz	Yes	No	Yes	Yes	Yes	Yes
Dong	Yes	Yes	Yes	Yes	Yes	Yes
Krishnan	Yes	Yes	Yes	Yes	Yes	Unclear
In't Veld	Yes	Yes	Yes	Yes	Yes	Unclear
Mundada	Yes	Yes	No	Yes	Yes	Unclear
Sanchez	Yes	No	Yes	Yes	Yes	Yes
Shin	Yes	Yes	Yes	Yes	Yes	Unclear
Terzi	Yes	Yes	No	Yes	Yes	Yes
